# Is the Invasive Species *Listronotus bonariensis* (Kuschel) (Coleoptera: Curculionidae) (Argentine Stem Weevil) a Threat to New Zealand Natural Grassland Ecosystems?

**DOI:** 10.3389/fpls.2016.01091

**Published:** 2016-07-26

**Authors:** Barbara I. P. Barratt, Diane M. Barton, Bruce A. Philip, Colin M. Ferguson, Stephen L. Goldson

**Affiliations:** ^1^AgResearch InvermayMosgiel, New Zealand; ^2^Better Border BiosecurityMosgiel, New Zealand; ^3^AgResearch LincolnChristchurch, New Zealand

**Keywords:** *Listronotus bonariensis* (Kuschel), invasive species, native grasses, *Chionochloa rigida*, *Festuca novae-zelandiae*, *Poa colensoi*, *Lolium perenne*

## Abstract

*Listronotus bonariensis* (Argentine stem weevil) is a stem-boring weevil that has become a major pasture pest in New Zealand, and cool climate turf grass in Australia. This species is also frequently found in native tussock grassland in New Zealand. Laboratory and field trials were established to determine the risk posed to both seedlings and established plants of three native grass species compared to what happens with a common host of this species, hybrid ryegrass (*L. perenne* X *L. multiflorum*). Adult weevil feeding damage scores were higher on *Poa colensoi* and *Festuca novae-zelandiae* than *Chionochloa rigida*. Oviposition was lower on *P. colensoi* than hybrid ryegrass, and no eggs were laid on *F. novae-zelandiae*. In field trials using the same four species established as spaced plants *L. bonariensis* laid more eggs per tiller in ryegrass in a low altitude pasture site than in ryegrass in a higher altitude site. No eggs were found on the three native grass species at the tussock sites, and only low numbers were found on other grasses at the low altitude pasture site. Despite this, numbers of adult weevils were extracted from the plants in the field trials. These may have comprised survivors of the original weevils added to the plants, together with new generation weevils that had emerged during the experiment. Irrespective, higher numbers were recovered from the tussock site plants than from those from the pasture site. It was concluded that *L. bonariensis* is likely to have little overall impact, but a greater impact on native grass seedling survival than on established plants.

## Introduction

Environmental impacts of invasive alien species have been shown to be varied, including native species extinction, changes in species richness and abundance, and alterations to food web interactions etc. (Blackburn et al., 2014). However, in some cases exotic species have minimal demonstrated impacts in new environments, or have indirect impacts that may not be immediately apparent (Brockerhoff et al., 2010). In general, but with some exceptions, exotic invertebrates have shown low impact on plants in New Zealand's natural ecosystems (Brockerhoff et al., 2010) possibly as a result of the high level of endemism of New Zealand native plants and their phylogenetic distance from host plants of many invasive plant pests.

Native grasslands in New Zealand provide a number of ecosystem services depending on their degree of modification (Mark et al., 2013). These include pollination, biological control, water, and soil conservation. They also provide the basis for education, ecotourism, and recreational services. For example, it has been demonstrated that water collection and retention by tall tussock species from fog can yield more fresh water than any other land use measured (Mark and Dickinson, 2008). Disturbance to natural grasslands such as burning, grazing, intensification of land use and weed invasion threaten their ability to provide ecosystem services, but little is known about the threat from invasive exotic invertebrates.

Several exotic species of Curculionidae have been recorded in New Zealand native grasslands (Barratt et al., 1998; Brockerhoff et al., 2010; Mark et al., 2013), but few have been recorded to be feeding or breeding on New Zealand native plants. Their presence in native grasslands may often be simply a case of vagrance, for example the lucerne weevil, *Sitona discoideus* Gyllenhal is a strong flyer with an annual dispersal phase (Goldson et al., 1984). This species has been collected at 1300 m altitude in the Waikaia Ecological Region (Dickinson et al., 1998), and at 2800 m altitude on the Inland Kaikoura Ranges (Phillips, unpublished). However, its hosts are restricted to species of *Medicago* spp. and *Trifolium* spp. (Vink and Phillips, 2007) and it is unlikely to have host plants in New Zealand's native flora. In contrast, a flightless, polyphagous, European weevil, *Otiorhynchus ovatus* L. which occurs in tussock grasslands in Central Otago (Barratt et al., 2009) might feed on some New Zealand native plants (Brockerhoff and Bain, 2000), but as yet there are no published records of this. Furthermore, this species, and three other *Otiorhynchus* spp. that are established in New Zealand, were not recorded on native plants sampled by Kuschel (1990).

The invasive weevil species that is perhaps most likely to have host plants amongst New Zealand's native grassland flora is the “Argentine stem weevil” (*Listronotus bonariensis* (Kuschel) (Coleoptera: Curculionidae) (Barratt et al., 2007). This species was first reported in New Zealand in the late 1927 (Marshall, 1937) but its introduction is likely to have been earlier in the twentieth century (Kuschel, 1972) and there may have been more than one introduction. The weevil has become so abundant throughout New Zealand that it has been long recognized as an agricultural pest (Kelsey, 1958). Further, it is a dispersive flier (Goldson et al., 1999), and is the most frequently found exotic species in tussock grassland (Barratt et al., 2007). It has been collected in Otago from remnant native shrubland (Derraik et al., 2001), tussock grasslands (Murray et al., 2003), and up to 1640 m on Coronet Peak, Otago. In New Zealand's predominantly ryegrass pastures the species can reach adult densities of 700 per m^2^) (Barker and Addison, 1993) which is vastly higher than in the “vega” or “mallines” type valleys which Lloyd (1966) has suggested is its center-of-origin. These alpine ecosystems are high-fertility, moist valley areas south of 39°S in the Andes (Squeo et al., 2006; Stewart, 2014) and are the habitat equivalent of New Zealand alpine grasslands, herbfields and cushion bogs (Wardle et al., 2001). Typical native species of the “mallines” alongside the *Juncaceae* and *Cyperacae* are grasses such as *Festuca pallescens, Poa lanuginose*, and *Hordeum comosom* (Gaitán et al., 2010). These malline grasses are in the same genera as some of New Zealand's introduced Gramineae including common cereals and pasture grasses (Morrison, 1938; Jacques, 1940; Doull, 1954; Kain and Barker, 1966). *L. bonarensis* can frequently be found in association with these plants. Whether there are any *Lolium* spp. native to South America seems uncertain, but it has been suggested that *Lolium rigidum lepturoides* could be. Although closely related to cosmopolitan *Festuca* spp., *Lolium* spp. was not introduced into New Zealand until the arrival of the early European pastoralists, probably in the early 1900s (Hunt and Easton, 1989). *L. bonariensis* is also a major pest of cool climate turf grasses in Australia (Hardy et al., 1979). On the balance though, it seems that *L. bonariensis* has been highly adventive and has acquired host plants that it did not evolve with, at least at the species level.

Having become established throughout New Zealand, by the early 1990s *L. bonariensis* was estimated to be causing damage to the pastoral sector amounting to NZ$78–251M annually (Prestidge et al., 1991). These estimated losses have been considerably offset by the successful introduction of the parasitoid, *Microctonus hyperodae* Loan (Hymenoptera: Braconidae) in 1992 (Goldson et al., 1993) combined with the widespread adoption of endophytic ryegrass cultivars which provide resistance to *L. bonariensis* (Popay and Wyatt, 1995). Recent research, however, has indicated that *M. hyperodae* is becoming less effective as a biological control agent for reasons that are not entirely understood (Popay et al., 2011; Goldson et al., 2014).

While the impact of *L. bonariensis* in intensive pastoral ecosystems is well understood, little is known of the potential risk posed by this species to New Zealand's natural grassland ecosystems, despite the species being so commonly encountered in this environment (Barratt et al., 2009; Brockerhoff et al., 2010). In this study, our objective was to determine the impact of *L. bonariensis* on tussock grasslands in Otago, New Zealand by focusing specifically on the effect of adult and larval feeding on seedling tussock plants both in the laboratory and amongst established plants in the field. Three endemic grass species, which are amongst the most common and widespread members of tussock grassland plant communities New Zealand Plant Conservation Network (2016), were selected for the study on the basis that any substantial reduction in vigor or distribution of these species would change the plant communities in which such changes occurred. Hybrid ryegrass, (*L. perenne* X *L. multiflorum*), known to support high densities of *L. bonariensis*, was included in the study for purposes of comparison with the tussock plants. In order to determine whether the impact of *L. bonariensis* was dependent upon environmental conditions, a field trials using the same plant species were carried out in both an elevated altitude tussock grassland environment, and in a lowland pastoral ecosystem.

## Materials and methods

The tussock grass species used in this weevil impact study were, snow tussock (*Chionochloa rigida*), fescue tussock (*Festuca novae-zelandiae*), and blue tussock (*Poa colensoi*). The attack rates and damage on these plants were compared to the impacts on the introduced pasture species and known host, the hybrid ryegrass cv. “Grasslands Manawa” (*L. perenne* X *L. multiflorum*). This cultivar was selected because it is endophyte-free and is known to be susceptible to *L. bonariensis* damage (Kelsey, 1958; Goldson, 1982; Gaynor and Hunt, 1983; Barker, 1989). Henceforth, for simplicity, this will be referred to as hybrid ryegrass. For laboratory seedling survival studies and weevil feeding/oviposition experiments, the plant species were greenhouse grown from local seed. For the field experiments, mature plants obtained from a commercial native plant nursery were used.

*L. bonariensis* adults were collected from lowland pasture near Mosgiel, New Zealand using a suction sampler (modified BlowerVac®). These were maintained in groups of 30–50 in laboratory cages (160 × 180 × 75 mm deep) with a fine gauze lid. They were provided with hybrid ryegrass grown in commercial potting mix in cell trays, each cell containing 6–8 plants. When the plants were 50–100 mm high, they were transferred with roots and soil intact into a small plastic bag sealed abound the base of the plants with a cable clip. Two such plant “packets” were placed in each cage. Water was supplied in the form of 4 soaked dental wicks placed in the cage. Weevils were kept for up to a week until required for experiments.

## Laboratory experiments

### Laboratory experiment (seedling survival)

**Figure 1:**
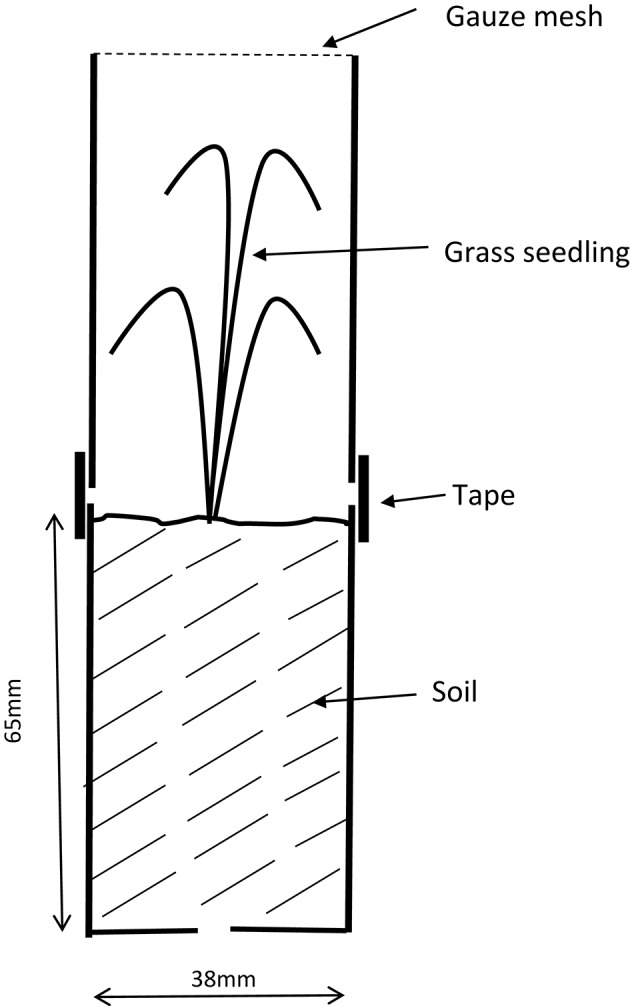
**Diagram to show container used for seedling damage experiment**.

Seed of *C. rigida, P. colensoi, F. novae-zelandiae* and hybrid ryegrass were sown in cell trays containing potting mix. After germination individual seedlings were transferred to 50 ml specimen containers (38 mm diameter × 65 mm high) filled with potting mix (Figure 1). When seedlings were 30–40 mm high three *L. bonariensis* adults were added to each container, which was then covered with another inverted 50 ml pot held in pace with tape. The lower container was provided with drainage holes and the upper container was closed at the top with fine mesh to prevent the escape of the weevils and reduce condensation. Thirty replicates of each grass species (15 for *C. rigida*) were established and they were exposed to the weevils for 18–20 days. An equivalent number of replicate plants of each species were also established as controls without weevils. Containers were spatially randomized in a screen house exposed to ambient outdoor temperatures. At the end of the exposure period plant survival was assessed and a feeding damage score was recorded on a scale of 1–5, where 1 was undamaged or very superficial feeding damage, and 5 was heavily damaged or almost completely consumed by weevil feeding. Plants were considered not to have survived if damage caused the plants to become desiccated and collapsed, or if feedings was so severe that no foliage remained.

It was not possible to conduct all experiments simultaneously because of different plant germination and growth rates. Thus experiments were carried out in both the summers of 2011–2012 and 2012–2013 in a screen house. Hybrid ryegrass was included in experiments carried out in both years. Control plants were left unexposed to weevils.

### Laboratory experiment (Weevil oviposition)

Seeds of the four grass species were grown as above until they were approximately 120 mm high with 2–3 tillers, depending upon the species. Plants were potted up individually into 38 mm diameter × 65 mm high containers filled with potting mix as above. Petri dishes were prepared with 85 mm diameter holes cut in their centers across which, fine nylon gauze was glued. Each pot's seedling leaves were then poked through the mesh and the Petri dishes gently lowered to the base of the plants such that the dish rested on top of the pots at soil level. This arrangement thus prevented weevils from moving into the soil while allowing the tillers to continue to grow through the mesh and allowing access for the weevils to the tiller bases. A transparent plastic cylinder (65 mm diameter; 140 mm high) with a fine mesh lid was placed over the plants and attached to the Petri dish base with Blutac®. Three *L. bonariensis* adults then were introduced to each container. All were supplied with a water-soaked dental wick, and left for 18–20 days in a screen house as above. At the end of this period the weevils were removed and dissected in order to determine gender. Replicates with no females were excluded from the analysis, leaving between 28 and 33 replicates of hybrid ryegrass, *P. colensoi* and *F. novae-zelandiae*, and 15 for *C. rigida*. Plant tillers were counted, the basal tiller diameters measured and then all tillers were examined under a binocular microscope for weevil eggs and larvae. As for the seedling survival study, this experiment was run over two seasons.

## Field trials

Two field study sites were used. The first was in a tussock grassland ecosystem on the East Otago Plateau between the Rock and Pillar Range and the Lammermoor Range along the Old Dunstan Road (−45.490, 169.964) and the second was in pasture on the Taieri Plain near Mosgiel (−45.862, 170.378). These are referred to as the “tussock grassland site,” and the “lowland pasture site” respectively.

Both sites were initially treated with glyphosate to prepare them for planting. At the tussock grassland site the foliage of any surviving *C. rigida* plants was trimmed to 50 mm. At both locations spaced mature plants of *C. rigida, P. colensoi, F. novae-zelandiae* and hybrid ryegrass were planted in rows 1m apart with 700 mm between the plants. These were arranged in four blocks each comprising 30 replicates of plant species randomly arranged. The site characteristics and treatment details are given in Table 1. The test plants were transplanted in the autumn of 2012 and were left to become established until the following early summer, when 10 *L. bonariensis* adults were introduced to each plant. At this time the plants were enclosed in gauze bags pushed into the soil with a metal cylinder.

**Table 1:** **Field trial site description and sample dates**.

**Site**	**Taieri Plain (lowland pasture site)**	**East Otago Plateau (tussock grassland site)**
Altitude above sea level	20 m	900 m
Mean annual rainfall	684 mm	630 mm
Soil type	Wingatui silt loam	Teviot silt loam
Vegetation	pasture	*Chionochloa rigida* grassland
Site prepared	Jan 2012	Dec 2011
Tussocks planted	3 Apr 2012	8 Mar 2012
*L. bonariensis* adults added	7 Dec 2013	12 Dec 2013
Tiller samples taken for eggs and larvae	15 Jan 2013	1 Feb 2013
Plants excavated	27 Mar 2013	2 May 2013

Five weeks after weevil introduction in the lowland pasture site and after 7 weeks in the tussock grassland site (Table 1), 5–10 tillers were selected from different parts of each test plant and cut near the tiller base. A longer period was allowed for oviposition and larval emergence at the cooler higher altitude tussock grassland site. The excised tillers were taken to the laboratory for eggs and larval counts. Based on a technique developed by Goldson (1978) larval counts were carried out by placing the tillers on a wire mesh over a 5l container containing glycerol into which the larvae dropped while escaping from the desiccating tillers. The glycerol was thereafter filtered through a fine mesh and the larvae counted. After 2 weeks when no further larvae were extracted, the tillers were stored in a deep freeze pending microscopic examination for eggs and any remaining larvae.

In late March 2013 (lowland pasture site) and early May 2013 (tussock grassland site) the entire plants were excavated to a depth of approximately 100 mm. In the laboratory, the foliage from the plants was cut from the base of each plant and both the foliar and basal parts of the plants (which included the root material) were placed in modified Tullgren funnels (Crook et al., 2004) for 7 days to extract adult and larval *L. bonariensis*. The funnels used heat from 4 × 150 W light bulbs positioned approximately 300 mm above each sample to extract all invertebrates, which were collected below the funnels into monopropylene glycol. After extraction, the collected material was washed in water through a fine sieve to retain the invertebrates which were stored in 70% ethanol, before examination under a binocular microscope to identify and count adult and larval stages of *L. bonariensis*. Adults were examined to determine whether they were the original weevils used in the experiment, or part of a new generation that had emerged during the experiment. This was assessed based on the degree of wear of scales on the elytra, the condition of hairs on the elytral surface and the presence of any teneral individuals.

## Data analysis

Laboratory and field data were analyzed using Genstat 16.2 (VSN International, 2013). Seedling survival data were analyzed using a binomial GLM with a logit link function to compare the effects of plant species. ANOVAs were carried out on the laboratory seedling damage scores, and field adult and larval density data to ascertain the significance of site and plant species treatments effects. For oviposition data a simple linear comparison of means was carried out and standard errors calculated. Comparisons for the laboratory data were made within but not between years. For the field data, the proportion of the adults extracted from the tussock plants at the end of the experiment were analyzed using a binomial GLM with a logit link function to compare the effects of plant species.

## Results

### Laboratory experiments—seedling survival and Weevil oviposition

The results of the laboratory seedling experiments are shown in Table 2. All control seedlings (not exposed to *L. bonariensis* adults) survived, and scored 1 (undamaged) for feeding damage so these data have not been included in Table 2.

**Table 2:** **Survival and damage scores of seedling grasses exposed to *L. bonariensis* adults in the laboratory**.

**Plant seedling survival**						
**Plant species**	**Date exposed**	**Date assessed**	**No plants**	**Mean plant damage score (1–5)**	**Mean proportion of plants surviving (SE)**	
Hybrid ryegrass	23 Dec 11	10 Jan 12	30	2.27 (0.31)	0.97 (0.03)	
*P. colensoi*	23 Dec 11	10 Jan 12	30	4.3 (0.20)	0.43 (0.09)	
Hybrid ryegrass	13 Dec 12	2 Jan 13	30	4.3 (0.24)	0.47 (0.09)	
*F. novae-zelandiae*	13 Dec 12	2 Jan 13	30	4.3 (0.09)	0.43 (0.09)	
*C. rigida*	13 Feb 12	2 Mar 12	15	2.53 (0.44)	0.8 (0.1)	
**Weevil Oviposition**						
**Plant species**	**Date exposed**	**Date assessed**	**No plants**	**Mean no. eggs plus larvae per female (SE)**	**Mean no. tillers per plant (SE)**	**Mean diam of tillers (SE)**
Hybrid ryegrass	23 Dec 11	11 Jan 12	28	9.89 (2.19)	3.1 (0.26)	1.36 (0.08)
*P. colensoi*	23 Dec 11	11 Jan 12	33	0.53 (0.19)	1.27 (0.09)	0.76 (0.05)
Hybrid ryegrass	13 Dec 12	2 Jan 13	30	2.47 (0.87)	2.37 (0.22)	1.6 (0.09)
*F. novae-zelandiae*	13 Dec 12	2 Jan 13	30	0	1.67 (0.15)	0.8 (0.04)
*C. rigida*	17 Feb 12	2 Mar 12	15	0.13 (0.13)	1.6 (0.25)	1.39 (0.11)

*Weevil oviposition on seedling grasses is also shown in relation to mean tiller numbers and diameter. The means for seedling damage and survival are those predicted by the regression model; standard errors are shown in brackets*.

In the experiments carried out in 2011 hybrid ryegrass seedling survival after 18–20 days exposure to adult *L. bonariensis* was significantly higher than for *P. colensoi* (*P* < 0.001), but in 2012 survival of hybrid ryegrass and *F. novae-zelandiae* survival were not significant (Table 2). The plant damage scores were accordingly higher for *P. colensoi* than hybrid ryegrass (*P* < 0.001) in 2011, but not different in the 2012 comparison between hybrid ryegrass and *F. novae-zelandiae* (Table 2). *C. rigida* exposed to *L. bonariensis* in February 2012 sustained a relatively low damage score and consequently survival was about 80%.

Oviposition was higher on hybrid ryegrass than on *P. colensoi* (*P* < 0.001) and *F. novae-zelandiae* (*P* < 0.01) in the 2011 and 2012 comparisons respectively. Small numbers of eggs were laid on *P. colensoi* but none were laid on *F. novae-zelandiae* (Table 2). Oviposition on *C. rigida* was not compared directly with hybrid ryegrass, but the number of eggs laid on this species was also very low. The hybrid ryegrass plants had more tillers than both *P. colensoi* and *F. novae-zelandiae* (*P* < 0.001) in both 2011 and 2012 experiments, and the mean tiller diameter of hybrid ryegrass was about twice that of both *P. colensoi* and *F. novae-zelandiae* (*P* < 0.001) when measured in 2011 and 2012 comparisons. *C. rigida* had a similar number of tillers to *F. novae-zelandiae*, but tiller width was more similar to hybrid ryegrass.

## Field trials

At both the lowland pasture site and the tussock grassland site, no eggs at all were found in tillers of the three native grass species. However, in hybrid ryegrass, eggs were found in tillers at both sites, with significantly more at the lowland pasture site (*P* < 0.001) (Table 3). Larvae were found in tillers of all species at the lowland pasture site, but numbers were very low in the native grass species. No larvae were found in tillers of the native grass species at the tussock grassland site. In hybrid ryegrass significantly more larvae were found at the lowland pasture site than the tussock grassland site (*P* < 0.001) (Table 3). An average of 2 immature stages (eggs plus larvae) per tiller was found in the hybrid ryegrass at the lowland pasture site whereas in the native grass species 0.01–0.03 immature stages per tiller were found in the native grass species (Table 3).

**Table 3:** **Mean numbers of eggs and larvae in tiller samples, and mean number of immatures per tiller taken from the field trials at the lowland pasture and tussock grassland sites**.

**Plant species**								
	**Lowland pasture site**				**Tussock grassland site**			
	***Chionochloa rigida***	***Festuva novae-zelandica***	***Poa colensoi***	***Lolium perenne***	***Chionochloa rigida***	***Festuva novae-zelandica***	***Poa colensoi***	***Lolium perenne***
Mean no. eggs in tillers	0	0	0	14.6 (2.4)	0	0	0	5.43 (0.96)
Mean no. larvae in tillers	0.1 (0.06)	0.3 (0.12)	0.13 (0.08)	5.47 (0.81)	0	0	0	0.69 (0.21)
Mean no. immatures per tiller	0.01 (0.01)	0.03 (0.01)	0.013 (0.01)	2 (0.28)	0	0	0	0.61 (0.11)
Mean no. adult weevils per plant	1.2 (0.27)	1.07 (0.34)	0.83 (0.22)	3.03 (0.46)	2.7 (0.42)	2.53 (0.31)	2.87 (0.34)	5.57 (0.58)
Proportion of new generation adults	0.30	0.15	0.08	0.64	0.11	0.06	0.07	0.14

*The mean number of adult weevils, and proportion of them which were “new” generation, extracted from plants at the end of the trials is also shown. Standard errors are given in brackets*.

When the plants were excavated and heat-extracted in the laboratory to recover adult weevils, some larvae were also collected. These comprised approximately 10 and 5% of the total numbers at the lowland pasture and tussock grassland sites respectively. The larval numbers were combined with adult counts for the analysis. There was a significant site effect with more adults and larvae recovered from the tussock grassland site (*P* < 0.001), and a significant plant species effect with more weevils recovered from hybrid ryegrass than the native grass species at both sites (*P* < 0.001). There was no significant site × plant species interaction (*P* = 0.461).

The assessment of adult weevils extracted to determine whether they were the original weevils added to the plants in the field, or new generation weevils that had developed through their life cycle during the experiment, showed that development was faster at the lowland pasture site as would be expected. Over 60% of the weevils collected from ryegrass here were new generation compared with 14% collected from ryegrass at the tussock grassland site (Table 3). This was true also for the other grass species except for *P. colensoi* where only 7–8% of weevils were new generation at both sites. Interestingly, despite no immature stages being found in native species at the tussock site, a very small number of apparently new generation weevils were extracted from these plants.

## Discussion

This study was designed to determine whether the invasive pest species, *L. bonariensis*is is likely to be having an ongoing impact on native grasses in natural grassland ecosystems, where it is commonly found. The data suggest that under current conditions, it is unlikely that this invasive weevil is posing a threat to the tussock species investigated, especially to mature plants.

Laboratory experiments which examined the survival of seedling stage native plants exposed to weevils indeed found that that two of the native grass species tested, *P. colensoi* and *F. novae-zelandiae*, were equally or more susceptible to feeding damage and mortality as hybrid ryegrass seedlings. Conversely snow tussock seedlings (*C. rigida*), were significantly less susceptible (Table 2). The difference between the hybrid ryegrass results for the two seasons is unclear, but the seedlings used in the second season might have been slightly smaller than those used in the first season. This is indicated by the smaller number of tillers present in the second season.

It was noted by Goldson (1982) that some grass species showing resistance to insect feeding, could have been the result of elevated alkaloid levels. This was later found to be associated with endophyte, which is now well known as conferring insect resistance to some ryegrasses (but absent from “Grasslands Manawa” used in this study) and some other pasture grass species (Rowan and Gaynor, 1986). Very little is known about endophytes in native grass species but a survey of 24 species (including several samples of *P. colensoi*, one of *F. novae-zelandiae* but not *Chionochloa*) revealed no endophytic associations (Rolston et al., 2002).

*Listronotus bonariensis* eggs were found to be absent in the native grasses at both sites, but presence of larvae in the tillers in the native grass species at the lowland pasture site (Table 3) indicated that any eggs laid had already hatched by the time of sampling. Furthermore, since the weevils that were recovered from the tussocks at both sites, comprised at least some new generation adults this indicated that some full development had occurred on the native plants. In contrast, at the time of sampling, eggs were still being found in hybrid ryegrass plants at both sites. This could indicate that a longer period of oviposition was associated with the more suitable host plant or that the scarcity of eggs being laid in the native plants was below the detection threshold. *L. bonariensis* prepares to oviposit by chewing a hole in the leaf sheath of a tiller and then via the hole, insert eggs between the upper and lower epidermis of the leaf sheath. When the larvae hatch they typically bore into the tiller and move downwards toward the base (Barker and Addison, 1990). This study has shown that *L. bonariensis* oviposition was significantly lower on native grasses compared with hybrid ryegrass. In *F. novae-zelandiae* there was no oviposition recorded in the laboratory or in the field, although in a preliminary laboratory study Lister (2006) found some oviposition on *F. novae-zelandiae*, but none on *C. rigida*. Goldson and Penman (1979) noted that females require a tiller diameter of about 1 mm for oviposition. Mean tiller diameter of the *P. colensoi* and *F. novae-zelandiae* were both found to be < 1 mm in this study, which could have contributed to the comparatively low (or lack of) oviposition in these species.

Goldson (1982) further hypothesized that, as well as a minimum tiller diameter, ovipositional preference and the typically low and highly variable levels of first to second instar survival (6–23%)were inversely proportional to plant cellulose levels. In this study cellulose levels were not measured in the native grasses, but Bailey and Connor (1972) measured cellulose levels of 30–32% of dry weight in *Chionochloa rigida*. In hybrid ryegrass, depending upon the cultivar, cellulose levels were recorded in the region of 10–15% of dry weight (Bailey, 1965), although Lancashire and Ulyatt (1975) measured levels between 18 and 27% depending upon cultivar and season. Native grasses also often accumulate dead tissue at the base of tillers, which might also impede weevil access to the tillers for oviposition. Conversely, tussock burning which is practiced to remove litter, and promote new palatable regrowth in tussock grasses for grazing animals, could possibly render the plants more palatable to *L. bonariensis*, and thereby facilitate oviposition. It needs to be recognized also that the weevils collected for the field experiment were sourced of necessity from lowland pasture, and hence feeding/oviposition preferences as well as general fitness for a native grassland environment might have been influenced by such conditioning. In future work it would be of interest to determine whether tussock grassland-sourced populations of *L. bonariensis* might have become adapted to native grass species and the environment in general.

Given the uncertainty around the taxonomic affiliations of the genera *Lolium* and *Festuca* spp., it is likely that the only new association with the native grass species in this study was *Chionochloa*; however, this was not found to be a highly susceptible to *L. bonariensis*, probably as a result of the tough stem epidermis.

The density of *L. bonariensis* used in this study's field sites was deliberately higher that normally encountered in native tussock grassland at 900 m so that a “worse-case scenario” could be investigated. Previous work has shown that *L. bonariensis* can reach densities of up to 40/m^2^ in lower altitude tussock grassland at 360 to 640 m, with a maximum density of 13/m^2^ at 450 to 850 m (Barratt et al., 2007). An intensively studied tussock grassland site at 1100 m yielded a January maximum of 5.5/m^2^ (Barratt et al., 2009). Climatic conditions are likely to be a factor in the fitness and performance of *L. bonariensis* in the tussock grassland environment, and almost certainly its phenology. Barker (1988) found that there was no egg or larval development at < 10°C in the laboratory. Temperature was not measured during this study but to provide some context, at a site at 460 m on the Lower Otago Plateau, 35 km from the tussock grassland site, the 30-year mean monthly temperature was 12–13°C in December and January with mean monthly mimina of 7–8°C respectively. This indicates development of *L. bonariensis* immature stages at the tussock grassland site would be more protracted, and might explain the higher number of adult weevils remaining at the tussock grassland site at the end of the field study and smaller proportion of teneral and new generation adults.

## Conclusions

This study has indicated that *L. bonariensis* is unlikely to be a significant threat in tussock grassland, at least to the very common species included in this investigation. If development times at this elevation are indeed a factor, then this might also apply to other native grass species occurring in this environment. For rare or endangered native grass species, the potential risk from this invasive weevil might be greater. Should the density of *L. bonariensis* increase, adult feeding could play a role in reducing seedling establishment for some native species. The impact of moderate animal grazing pressure on *C. rigida* seedling survival (Lee et al., 1993) is known to be significant (Lee et al., 1993). The relatively minor mortality rates found in *C. rigida* seedlings exposed to *L. bonariensis* for 2–3 weeks is trivial in comparison. It is, therefore, unlikely that *L. bonariensis* would add substantially to the overall impact of grazing animals. However, the other grass species tested could possibly be at more risk than *C. rigida* based on indications of greater feeding damage and reduced plant survival. Irrespective though the climatic conditions in higher altitude tussock grasslands, combined with the relative unsuitability of native grass species to *L. bonariensis* all point to little overall impact.

## Author contributions

BB led the project design, data collection and analysis, writing up and manuscript submission. DB assisted with planning the project, carried out a large proportion of the field and laboratory work and assembled data for analysis. BP carried out a large proportion of the field and laboratory work and assisted with assembly of data for analysis. CF assisted with planning the project led the field site preparation for the trial, and field work data collection. SG provided background information on the biology and ecology of Argentine stem weevil, both in South America and in New Zealand, and had a major input into data interpretation and writing the manuscript.

## Funding

The Australasian Congress of Grassland Invertebrate Ecology provided financial assistance for open access publication fees.

### Conflict of interest statement

The authors declare that the research was conducted in the absence of any commercial or financial relationships that could be construed as a potential conflict of interest.
